# Declarations on euthanasia and assisted dying

**DOI:** 10.1080/07481187.2017.1317300

**Published:** 2017-05-09

**Authors:** Hamilton Inbadas, Shahaduz Zaman, Sandy Whitelaw, David Clark

**Affiliations:** ^a^ School of Interdisciplinary Studies, University of Glasgow, Glasgow, Scotland, UK

## Abstract

Declarations on end-of-life issues are advocacy interventions that seek to influence policy, raise awareness and call others to action. Despite increasing prominence, they have attracted little attention from researchers. This study tracks the emergence, content, and purpose of declarations concerned with assisted dying and euthanasia, in the global context. The authors identified 62 assisted dying/euthanasia declarations covering 1974–2016 and analyzed them for originating organization, geographic scope, format, and stated viewpoint on assisted dying/euthanasia. The declarations emerged from diverse organizational settings and became more frequent over time. Most opposed assisted dying/euthanasia.

Euthanasia and certain forms of assisted dying are currently legal or decriminalized in just a few countries. The Netherlands (2001), Belgium (2002), and Luxembourg (2009) have legalized euthanasia (Cohen, Van Landeghem, Carpentier, & Deliens, 2014), and Canada (2016) has introduced a federal law allowing medical aid in dying (Chochinov & Frazee, 2016; Upshur, 2016). In these four countries, euthanasia/assisted dying is legal provided those involved follow certain procedures involving an informed and competent request. By long standing arrangement, Switzerland does not prosecute those who assist a suicide death, provided they do not benefit from the outcome. A similar, more recent arrangement prevails in Colombia.

In the United States, some individual states have legalized physician assisted suicide (PAS) (Varadarajan, Freeman, & Parmar, 2016). This process involves a doctor prescribing lethal drugs to a person who, following defined procedures, wishes to die by taking the drugs, and then does so. Oregon legalized PAS in 1997 and subsequently so did Washington State, Montana, Vermont and California (Gostin & Roberts, 2016).

The case for assisted dying and/or euthanasia is being debated in many settings, especially those where no specific legislation yet exists and has led to a range of advocacy interventions. One way to influence policy is by generating formal statements on single issues. When associations, organizations and groups concerned about end-of-life issues promulgate their views on a specified matter, they can draw it to public attention and call for change.

We refer to advocacy interventions of this type as *declarations*. Although they may take different names (statement, resolution, manifesto, charter, commitment, or proclamation) such declarations group around a common purpose. They capture the goals of interest groups, make statements of intent, point to a more desirable state of affairs, and encourage greater awareness to achieve a stated goal. These declarations have no legal mandate but do have potential for influencing laws, policies, systems and processes on end-of-life issues. They have become a part of the landscape of end-of-life care, and the debates that swirl around it.

At the same time, they are poorly documented and largely ignored by researchers. Yet they are important markers in the evolution of end-of-life discourse. They give perspective on the changing discussion around specific issues and have some importance within the culture of many end-of-life care organizations. They merit research scrutiny, in particular, when declarations on the same topic take up opposing or differing perspectives.

Building on an earlier study of declarations in support of palliative care development (Inbadas, Zaman, Whitelaw, & Clark, 2016), we focus here on such statements as they relate to euthanasia and assisted dying. Our aims were to (a) track over time the emergence of euthanasia/assisted dying declarations, in the global context, (b) describe their form, structure and characteristics, and (c) document their stated purposes. We set out to build a comprehensive collection of declarations that relate to euthanasia/assisted dying and are available in the public domain.

## Method

First, during the period August to December 2016 we identified euthanasia/assisted dying declarations, using English language searches on the Google search-engine with the key words *euthanasia, assisted dying*, and *assisted suicide*, in combination with *declaration, charter, manifesto, resolution*, and *statement*. This process generated 57 declarations before reaching saturation.

Second, we searched websites of key euthanasia/assisted dying organizations and palliative care associations. This process generated 16 declarations from the websites of the World Federation of Right to Die Societies, the International Association for Hospice and Palliative Care, and Dying with Dignity Canada.

We then assessed these 73 declarations for inclusion in the study. We included all declarations that comprised formal public statements and contained at least one element of advocacy (Dunning & Lloyd, 1995). Using this formulation, we excluded 11 declarations. These comprised eight statements from individual hospices explaining their position on euthanasia/assisted dying; one response of a political party to an individual who had asked for the party’s position on the Supreme Court decision regarding assisted dying; and two detailing implications for pharmacists and nurses (respectively) if euthanasia/assisted dying were legalized.

We then subjected the remaining 62 declarations (Table 1) to content analysis, with the following objectives, to (a) build a timeline of their publication, (b) identify the organization/association that issued them, (c) record the stated viewpoint on euthanasia/assisted dying, (d) assess the geographical scope of the declarations, (e) determine their format and structure, and (f) document their recommendations. We chose content analysis because it is applicable for the analysis of text from a variety of documents and facilitates the study of their characteristics (Duncan, 1989) and is appropriate for exploring areas of study that lack pre-existing theoretical frames (Ruiz Ruiz, 2009). Our categories were year of publication, geographical scope, formats, types of organization, and viewpoints expressed in the declarations. We also analyzed the relationship between different categories (e.g., between the timeline and viewpoint expressed, and type of organization and viewpoint expressed).

**Table 1.** Sixty-two declarations on euthanasia/assisted dying.

Year	Title of declaration	Organization (type)	Geographical scope	Position on euthanasia/ assisted dying	Key content	Source
1974	A Methodist Statement on Euthanasia	The Methodist Church (Religious)	Global	Against	Better end-of-life care; The need is not so much to change the law but to alter the attitude of society towards death.	http://www.methodist.org.uk/downloads/pi_euthanasia_74.pdf
1976	Tokyo declaration of August, 1976	The World Federation of Right to Die Societies (Pro-euthanasia/assisted dying)	Global	For	The “Living Will” should be made legally effective, and pursuant to this, efforts toward its legalization should be made.	http://www.worldrtd.net/news/tokyo-declaration-august-1976
1980	Declaration on Euthanasia	Sacred Congregation for the Doctrine of the Faith (Religious)	Global	Against	Those who work in the medical profession ought to neglect no means of making all their skill available to the sick and dying; but they should also remember how much more necessary it is to provide them with the comfort of boundless kindness and heartfelt charity.	http://www.vatican.va/roman_curia/congregations/cfaith/documents/rc_con_cfaith_doc_19800505_euthanasia_en.html
1987	WMA Declaration on Euthanasia	World Medical Association (Health care)	Global	Against	Euthanasia even at their own request is unethical; physicians should respect the desire to allow natural death.	http://www.wma.net/en/30publications/10policies/e13/
1988	Euthanasia Ethics Statement	Christian Medical and Dental Association (Religious)	Global	Against	While rejecting euthanasia, we encourage the development and use of alternatives to relieve suffering, provide human companionship, and give opportunity for spiritual support and counselling.	http://cmda.org/library/doclib/Euthanasia-with-References.pdf
1991	Euthanasia Statement	National Conference of Catholic Bishops (Religious)	National (U.S.)	Against	Reject proposals to legalize euthanasia, families to discuss issues surrounding the care of terminally ill loved ones in light of sound moral principles and the demands of human dignity. (Health care) professionals, legislators, and all involved in this debate, to respect the inherent worth of all human beings.	http://www.usccb.org/issues-and-action/human-life-and-dignity/end-of-life/euthanasia/statement-on-euthanasia-1991.cfm
1992	WMA Statement on Physician-Assisted Suicide	World Medical Association (Health care)	Global	Against	PAS is unethical. However, the right to decline medical treatment is a basic right.	http://www.wma.net/en/30publications/10policies/p13/
1992	Resolution On Euthanasia And Assisted Suicide	The Southern Baptist Convention (Religious)	National (U.S.)	Against	Scientists and physicians to continue their research into more effective pain management; we oppose efforts to designate food and water as “extraordinary treatment,“; we reject as appropriate any action which, of itself or by intention, causes a person’s death; we call upon federal, state, and local governments to prosecute under the law physicians or others who practice euthanasia or assist patients to commit suicide.	http://www.sbc.net/resolutions/493
1992	Physician Assisted Suicide	The Christian Medical & Dental Associations (Religious)	Global	Against	In order to affirm the dignity of human life, we advocate the development and use of alternatives to relieve pain and suffering, provide human companionship, and give opportunity for spiritual support and counselling.	https://cmda.org/resources/publication/physician-assisted-suicide-ethics-statement
1993	Statement On Euthanasia	Michigan Catholic Conference (Religious)	Global	Against	The medical and legal professions to study the issue with discipline, integrity and compassion; we call upon all people of good will to reflect on the value of life and its ultimate meaning; media not to capitalize on people’s confusion, ambivalence and even fear about the use of modern life-prolonging technologies, but to provide thorough research to clarify and enlighten.	http://www.micatholic.org/advocacy/board-bishops-statements/board-statements/statement-on-euthanasia/
1994	Hospice and Palliative Nurse Association’s Statement in Response to Supreme Court Ruling on Physician-Assisted Suicide	Hospice and Palliative Nurse Association (Health care)	National (U.S.)	Against	Support all public policy changes that would ensure access to hospice care.	http://hospicecare.com/resources/ethical-issues/statements-on-euthanasia-and-physician-assisted-suicide/
1994	Opinion 2.21—Euthanasia	American Medical Association (Health care)	Global	Against	Instead of engaging in euthanasia, physicians must aggressively respond to the needs of patients at the end of life.	http://www.ama-assn.org/ama/pub/physician-resources/medical-ethics/code-medical-ethics/opinion221.page?
1995	Voluntary active euthanasia – position statement of the Australian Association for Hospice and Palliative Care Inc.	The Australian Association for Hospice and Palliative Care (Health care)	National (Australia)	Against	Voluntary Active Euthanasia (VAE) is the deliberate action to terminate life. Refusal or withdrawal of futile treatment is not VAE. Public interest in VAE reflects a concern about lack of adequate support for people who are dying.	http://hospicecare.com/resources/ethical-issues/statements-on-euthanasia-and-physician-assisted-suicide/#AAHPC
1996	Resolution on assisted suicide	The World Federation of Right to Die Societies (Pro-euthanasia/assisted dying)	Global	For	The philosophy of hospice care neither hastens nor postpones death; does not support the legalization of voluntary euthanasia or assisted suicide; supports improved access to hospice care for terminally ill patients and their families.	http://hospicecare.com/resources/ethical-issues/statements-on-euthanasia-and-physician-assisted-suicide/
1996	The Melbourne Declaration on Physician Assisted Suicide	Southern Baptist Convention (Religious)	National (U.S.)	Against	We want to be able to respond compassionately without the risk of legal prosecution. We are convinced that suitable guidelines to protect all patients can be developed.	https://www1.umn.edu/humanrts/instree/melbourne.html
1996	Resolution On Assisted Suicide	National Hospice Organization, USA (Health care)	National (U.S.)	Against	Affirm the biblical and Hippocratic prohibitions against assisted suicide; encourage medical science in its efforts to improve pain management techniques; vigorously denounce assisted suicide as an appropriate means of treating suffering; vigorously denounce assisted suicide as an appropriate means of treating suffering.	http://www.sbc.net/resolutions/278
1997	Voluntary Euthanasia: The Council’s View	National Council for Hospice and Specialist Palliative Care Services (Health care)	National (England, Wales and Northern Ireland)	Against	strongly commend the development and growth of palliative care services in hospices, in hospitals and in the community; rejecting euthanasia as an option for the individual entails a compelling social responsibility to care adequately for those who are elderly, dying or disabled.	http://hospicecare.com/resources/ethical-issues/statements-on-euthanasia-and-physician-assisted-suicide/#AAHPC
1997	Resolution opposing the legalization of physician assisted suicide	Association for Persons with Severe Handicaps (Health care)	National (U.S.)	Against	The Association for Persons with Severe Handicaps opposes the legalization of Physician-Assisted Suicide.	http://www.independentliving.org/docs6/tash199712.html
1998	Zurich Declaration on Assisted Dying	The World Federation of Right to Die Societies (Pro-euthanasia/assisted dying)	Global	For	Excellent palliative care should not exclude the right to choose assisted dying.	http://www.patientsrightscouncil.org/site/zurich-declaration/
2000	The Boston Declaration on Assisted Dying	The World Federation of Right to Die Societies (Pro-euthanasia/assisted dying)	Global	For	“Terminal sedation” is the same as physician assisted dying; We urge other medical professionals, worldwide, to be more open about this form of physician assisted dying	http://www.worldrtd.net/news/boston-declaration-assisted-dying
2001	APA Resolution on Assisted Suicide	American Psychological Association (Health care)	National (U.S.)	Neutral	Encourage practicing psychologists to obtain training and engage in research on PAS.	http://www.apa.org/about/policy/assisted-suicide.aspx
2002	WMA Resolution on Euthanasia	World Medical Association (Health care)	Global	Against	Strongly encourage physicians to refrain from participating in euthanasia, even if national law allows it	http://www.wma.net/en/30publications/10policies/e13b/
2002	The Brussels Declaration on Assisted Dying	The World Federation of Right to Die Societies (Pro-euthanasia/assisted dying)	Global	For	We strongly believe that this fundamental choice should be extended, as soon as possible, to other areas of the world, as in Belgium, The Netherlands, Switzerland and Oregon	http://www.worldrtd.net/news/brussels-declaration-assisted-dying
2004	The Tokyo declaration	The World Federation of Right to Die Societies (Pro-euthanasia/assisted dying)	Global	For	Follow directives from patients even when the choices made by the patient lead to what may be an unintentionally induced hastened death	http://www.worldrtd.net/it/news/tokyo-declaration
2005	Resolution and Commentary on Physician Assisted Suicide	National Hospice & Palliative Care Organization (Health care)	National (U.S.)	Against	Commitment to the value of life and to the optimization of the quality of life; support improved knowledge of and access to hospice and palliative care for terminally ill people and their families; do not support the legalization of physician assisted suicide.	http://www.nhpco.org/sites/default/files/public/PAS_Resolution_Commentary.pdf
2006	Manifesto	The World Federation of Right to Die Societies (Pro-euthanasia/assisted dying)	Global	For	All competent adults who are suffering unbearably from incurable illnesses should have the possibility of various choices at the end of their life. The voluntarily expressed will of individuals, should be respected as an expression of intrinsic human rights.	http://www.worldrtd.net/manifesto
2007	Resolution on Assisted Dying	The United Reformed Church (Religious)	National (U.K.)	Against	Oppose any change in the law to permit voluntary euthanasia or assisted suicide; palliative treatment can also hasten death, we believe this to be acceptable when the intention of the treatment is pain relief and comfort of the patient; Living Will or Advance Directive can be helpful, however they should not be used to facilitate a person’s death.	http://www.cte.org.uk/Publisher/File.aspx?ID=136874
2009	Liberty and Death: A manifesto concerning an individual’s right to choose to die	Euthanasia Research & Guidance Organization (ERGO) (Pro-euthanasia/assisted dying)	Global	For	Medically hastened death by request should be made lawful; ‘suicide’ should no longer be a crime; it is unacceptable to prosecute well-meaning people for ‘assisted suicide’.	http://www.finalexit.org/liberty_and_death_manifesto_right_to_ die_by_derek_humphry.html
2011	The Dangers of Euthanasia: A Statement from the New Zealand Catholic Bishops	The Catholic Church in Aotearoa New Zealand (Religious)	National (New Zealand)	Against	Ensure that there are adequate resources for palliative care	http://www.catholic.org.nz/news/fx-view-article.cfm?ctype=BSART&loadref=51&id=239
2011	Physician-Assisted Suicide—BGS Position Statement	British Geriatrics Society (Health care)	National (U.K.)	Against	Urges improvement in the medical and social care of older people.	http://www.bgs.org.uk/index.php/consultations/835-psnstatementassistedsuicide
2011	HPNA Position Statement—Legalization of Assisted Suicide	Hospice and Palliative Nurses Association (Health care)	National (U.S.)	Against	Support public policy that ensures access to hospice and palliative care for persons facing the end of life; advise nurses practicing in states where assisted suicide is legal that they have the moral and legal right to refuse to be involved in the care of patients requesting assisted suicide.	http://www.irisproject.net/images/HPNA_Legalization_of_Assisted_Suicide_Position_ Statement_080311.pdf
2012	Statement on issues related to end-of-life care	The College of Family Physicians of Canada (Health care)	National (Canada)	Neutral	All Canadians—regardless of age, disease, stage of disease, and geographical location—should have access to palliative care that meets national standards	http://www.cfpc.ca/uploadedFiles/Resources/Resource_Items/Health_Professionals/CFPC%20Position%20Statement_Palliative%20Care_ENGLISH.pdf
2012	Statement on hospice care and assisted dying	Hospice New Zealand (Health care)	National (New Zealand)	Against	Improving access to hospice and palliative care services.	http://www.hospice.org.nz/cms_show_download.php?id=570
2012	Position Statement: Physician-Assisted Suicide	The Arc of the United States (Health care)	National (U.S.)	Against	Appropriate medical or palliative care to reduce and/or eliminate pain and discomfort can and must be provided	http://www.thearc.org/document.doc?id=3632
2012	Joint Resolution opposing physician-assisted suicide	Americans United for Life (Anti- euthanasia/assisted dying)	National (U.S.)	Against	Strongly opposes and condemns physician-assisted suicide.	http://www.aul.org/wp-content/uploads/2012/11/Joint-Resolution-Opposing-PAS-2013-LG.pdf
2013	Statement on Assisted Suicide	New Mexico Conference of Catholic Bishops (Religious)	Regional (New Mexico)	Against	Healthcare providers must make every effort to ensure that the available medications to eliminate or control pain are provided to a patient.	http://www.archdiocesesantafe.org/ABSheehan/Bishops/BishStatements/13.12.13PRNMCCBAssistedSuicideFinal.pdf
2013	Positional Statement: Euthanasia and Assisted Dying	The Salvation Army International (Religious)	Global	Against	Optimal pain control and the overall comfort of the individual;	http://www.salvationarmy.org/ihq/ipseuthanasia
2013	Position Statements: Euthanasia, Assisted Suicide, and Aid in Dying	American Nurses Association (Health care)	National (U.S.)	Against	Increase communication skills education; outreach to the media—public education about palliative care/dispel misunderstandings.	http://www.nursingworld.org/MainMenuCategories/EthicsStandards/Ethics-Position-Statements/Euthanasia-Assisted-Suicide-and-Aid-in-Dying.pdf
2013	Position Statement: The practice of euthanasia and assisted suicide	The Australian and New Zealand Society of Palliative Medicine (Health care)	International (Australia, New Zealand)	Against	The Palliative Medicine discipline does not include the practice of euthanasia; Patients have the right to refuse life sustaining treatments including the provision of medically assisted nutrition and/or hydration. Palliative sedation for the management of refractory symptoms is not euthanasia	http://palliativecarewa.asn.au/site/wp-content/uploads/2014/03/ANZSPM-Position-Statement-on-Euthanasia-and-Assisted-Suicide.pdf
2014	RCN Position statement on assisted dying	Royal College of Nursing (Health care)	National (U.K.)	Neutral	The RCN moved from opposing assisted dying to a position where the College neither supports nor opposes a change in the law to allow assisted dying. We believe that this position rightly reflects our members differing views on the issue.	https://www2.rcn.org.uk/__data/assets/pdf_file/0007/598876/RCN_Position_statement_on_assisted_dying_final.pdf
2014	Position statement on hospice care and assisted dying (assisted suicide) and recommendations	Hospice UK (Health care)	National (U.K.)	Against	Improving access to hospice and palliative care services should be a priority for governments around the UK.	http://www.ashgatehospicecare.org.uk/about-us/assisted-dying-bill/
2015	NDP Response: Physician-Assisted Dying	New Democratic Party (Political)	National (Canada)	Neutral	NDP government would draw from the highly-effective, consensual and broadly supported process undertaken by the Quebec government.	https://d3n8a8pro7vhmx.cloudfront.net/dwdcanada/pages/142/attachments/original/1440800177/Dying_with_Dignity_-_EN_-_081415.pdf?1440800177
2015	Joint Statement on the Assisted Dying (No. 2) Bill, 2015–16	The National Council for Palliative Care, Association for Palliative Medicine & National Palliative Care Nurse Consultants Group (Health care)	National (U.K.)	Against	Ensure round the clock access to high quality palliative care for people who are terminally ill; the responsibility for the decision to allow someone’s life to be ended prematurely should rest with the courts, with clinicians providing factual information only; provision of lethal interventions should not become part of palliative or other clinical care services.	http://www.ncpc.org.uk/sites/default/files/Joint%20statement%20on%20assisted%20dying%20bill_Final%20PDF.pdf
2015	Physician Assisted Death	Liberal Party of Canada (Political)	National (Canada)	Neutral	A Liberal government will appoint a committee to consider the ruling: The Supreme Court of Canada’s decision to strike down the ban on physician-assisted death.	https://d3n8a8pro7vhmx.cloudfront.net/dwdcanada/pages/142/attachments/original/1440800180/Dying_with_Dignity_LPC_Statement_(2015-08-26)_EN.pdf?1440800180
2015	Declaration Against Euthanasia and Assisted Suicide	The Canadian Conference of Catholic Bishops and The Evangelical Fellowship of Canada (Religious)	National (Canada)	Against	Fundamental difference between killing a person and letting her or him die of natural causes.	http://www.euthanasiadeclaration.ca/declaration/
2015	Assisted suicide	Christian Medical Fellowship (Religious)	National (U.K.)	Against	The patient’s ‘right to die’ would impose on the doctor a duty to assist.	http://www.cmf.org.uk/resources/publications/content/?context=article&id=26327
2015	Public Briefing on the APM’s Position on Assisted Suicide	The Association for Palliative Medicine of Great Britain and Ireland (Health care)	National (U.K.)	Against	For the vulnerable, dying, laws that make doctors the decision makers are unsafe; licensing doctors explicitly to prescribe or administer lethal drugs is not health care and must remain distinct.	http://apmonline.org/wp-content/uploads/2015/07/AS-Full-briefing-final.pdf
2016	Retired ANA Position Statement: Assisted Suicide	American Nurses Association (Health care)	National (U.S.)	Against	Nurses to understand, learn and act—compassionate and appropriate end-of-life care.	http://www.nursingworld.org/MainMenuCategories/EthicsStandards/Ethics-Position-Statements/prtetsuic14456.html?css=print
2016	Statement of the Catholic Bishops of Alberta on Assisted Suicide and Euthanasia	The Catholic Bishops of Alberta (Religious)	National (Canada)	Against	We ask our provincial government to undertake a consultation process open to any and all who wish to speak to the issue.	https://www.catholicculture.org/culture/library/view.cfm?recnum=11176
2016	Physician Assisted Suicide	Royal Australian and New Zealand College of Psychiatrists (Health care)	International (Australia, New Zealand)	Neutral	Psychiatric assessment and treatment should be considered for patients who request PAS of their doctors.	https://www.ranzcp.org/Files/Resources/College_Statements/Position_Statements/PS-67-Physician-Assisted-Suicide-Feb-2016.aspx
2016	International Association for Hospice and Palliative Care Position Statement: Euthanasia and Physician-Assisted Suicide	International Association for Hospice and Palliative Care	Global	Against	No country or state should consider the legalization of euthanasia or PAS until it ensures universal access to palliative care services and to appropriate medications, including opioids for pain and dyspnea.	http://online.liebertpub.com/doi/full/10.1089/jpm.2016.0290
	Salvation Army Euthanasia Statement	Salvation Army, New Zealand (Religious)	National (New Zealand)	Against	Encourages all New Zealanders to examine the proposed legislation carefully.	http://www.nathaniel.org.nz/press-releases/241-salvation-army-euthanasia-statement
	Position Statement on Euthanasia	Association for Medical and Therapeutic Self-Determination (Health care)	National (The Netherlands)	Against	We consider euthanasia – the termination of another person’s life—inadmissible, regardless of the circumstances.	http://metzelf.info/articles/Euthanasia2.html
	Position Statement on Assisted Suicide	UK Disabled People’s Council (Health care)	National (U.K.)	Against	We urge the government to start addressing the lack of adequate support, equality and justice.	http://www.ukdpc.net/site/position-statementsbr/assisted-suicide
	Death with Dignity: Legalizing Medically-Assisted Death	National Women’s Liberal Commission (Political)	National (Canada)	For	Voluntary medically-assisted death to be de-criminalized after a public consultation process; work with medical community to de-criminalize medically-assisted death in Canada.	https://www.liberal.ca/policy-resolutions/165-death-dignity-legalizing-medicallyassisted-death/
	The Assisted Dying Bill	The Patients Association	National (U.K.)	Neutral	If becomes legal: strict safeguards; no pressure on healthcare institutions to participate.	http://www.patients-association.org.uk/wp-content/uploads/2015/03/PA-Position-Statement-Assisted-Dying.pdf
	Manifesto for a dignified and natural end of life and promotion of quality health care in Quebec	The Living with Dignity network	Regional (Quebec, Canada)	Against	We call upon our fellow citizens to mobilize and pressure the governing bodies to improve existing palliative care, to ensure that all Quebec citizens end their lives naturally, surrounded by attention and affection.	http://vivredignite.org/en/manifesto/
	End of Life and Assisted Suicide – Our Policy Statement	Parkinson’s UK (Health care)	National (U.K.)	Neutral	People with Parkinson’s, their carers and families should be able to exercise their right to access effective health and social care services at every stage of the condition; should always involve timely provision of good quality information.	http://www.parkinsons.org.uk/content/end-life-and-assisted-suicide-our-policy-statement
	Declaration of Hope USA	Euthanasia Prevention Coalition (Anti- euthanasia/assisted dying)	National (U.S.)	Against	Pain control and palliative medicine should be given a higher priority in medical training.	http://declarationofhope.net/
	Declaration of Hope Canada	Euthanasia Prevention Coalition (Anti- euthanasia/assisted dying)	National (Canada)	Against	Pain control and palliative medicine should be given a higher priority in medical training.	http://declarationofhope.ca/
	Care Not Killing Declaration	Care Not Killing (Anti- euthanasia/assisted dying)	National (U.K.)	Against	Improve provision of good palliative care.	http://www.carenotkilling.org.uk/declaration/
	Canadian Palliative Care Association	Canadian Palliative Care Association (Health care)	National (Canada)	Against	Palliative care services to be accessible to all dying persons in Canada; people have the right at any time to refuse or stop treatment.	http://hospicecare.com/resources/ethical-issues/statements-on-euthanasia-and-physician-assisted-suicide/#AAHPC

## Results

We could identify the year of publication for only 51 declarations. The oldest declaration was issued at the Annual Conference of the Methodist Church of Great Britain in 1974. The next two decades saw only occasional examples. Between 1992 and 2009, 23 declarations were issued, and there were 23 more between 2011 and 2016.

The type of organizations producing these 62 declarations varied widely. Health care organizations (29) included national and international medical and nursing associations, specific fields of medicine such as palliative care or geriatric care, and societies representing particular patient groups, such as the Association for Persons with Severe Handicaps, Parkinson’s UK and The Arc of the United States. Religious organizations (16) were all Christian in orientation, including Methodist, Baptist, Catholic, the Salvation Army, the Reformed Churches, and the Christian Medical and Dental Association. Others included political parties (three) and those organizations instituted to advocate for (eight) or against (four) euthanasia/assisted dying.

Seven out of the eight declarations in the group established to advocate for euthanasia/assisted dying were issued by the World Federation of Right to Die Societies. The first of these was in 1976 and the remaining six were issued between 1996 and 2006 at 2-year intervals, corresponding with the biannual conferences of the Federation.

Nearly three quarters of the declarations (45/62) were against euthanasia/assisted dying and were issued by associations of palliative care and other health care disciplines, associations of patient groups, and churches. Nine declarations advocated for the introduction of euthanasia/assisted dying, of which seven were issued by the World Federation of Right to Die Societies. Among the eight declarations that expressed a neutral position, two were from political parties calling for further discussion on the subject. Others included health care associations representing divided views of members, organizations that expressed their commitment to equal treatment of all patients irrespective of their position on euthanasia, and those that refrained from taking a position because euthanasia/assisted dying was illegal in their respective countries.

All declarations issued by religious organizations were against euthanasia/assisted dying. Among health care organizations, 24 were against and 5 were neutral. Two declarations from political parties took a neutral position and one was for euthanasia/assisted dying.

Analyzing the 51 declarations where the year of publication could be identified, we found different viewpoints showing prominence over specific periods of time. The first two declarations from 1974 (against) and 1976 (for) represented either side of the argument. With two exceptions, all declarations issued in the 1990s were against legalizing euthanasia/assisted dying. Five out of the nine declarations published between 2000 and 2010, were in support. The period from 2011 to 2016, which showed highest activity (23), was dominated by declarations against euthanasia/assisted dying (18). The first declaration with a neutral stance appeared in 2001 and, after a break of 10 years, five declarations were issued between 2012 and 2016.

The majority of declarations (39) were oriented to national audiences: United States (14), United Kingdom (12), Canada (eight), New Zealand (three), and the Netherlands (one). Many were published because of a proposed change in legislation or a judicial decision. The international declarations (19) were all issued by organizations or churches with a global presence, such as the World Medical Association, The World Federation of Right to Die Societies, The Christian Medical and Dental Associations, The Salvation Army International, and the Sacred Congregation for the Doctrine of Faith. Two declarations involved two countries only (Australia and New Zealand), and two involved a specific region within a country (Quebec, Canada and New Mexico, United States).

The 62 declarations came in several formats. Most common was a statement of convictions (38) expressing beliefs and opinions. Others made recommendations (23) to governments, policy makers, health care professionals and the wider public, expressed specific concerns (10), made a call to action (seven) for governments, health institutions or the public, made an explicit position statement (six) of the organizations’ stand, described their action plan (three), and recorded their commitment to a cause or an aspect of care (two). Many declarations contained more than one of these formats.

Most declarations indicated the ethical or practical reasons for their position on euthanasia/assisted dying (Table 2) and included religious beliefs, moral standards of medical practice, and potential for the abuse of legalized assisted suicide and the right to die.

**Table 2.** Arguments for and against euthanasia/assisted dying expressed in declarations (*n* = 62).

For	Against
Autonomy	Sanctity of human life, life is a gift from god
Right to die with dignity	Religious prohibition “Thou shalt not kill”
Physicians’ responsibility for eliminating suffering and promoting dignified end of life	No right to kill
	Responsibility to protect life
	Vulnerable populations may be forced to end their lives
	In conflict with basic principles of medical/nursing practice

The recommendations in the declarations varied in relation to the “viewpoint” adopted: for, against or neutral. Recommendations from declarations for euthanasia or assisted dying included decriminalization of voluntary medically assisted death; legalizing medically hastened death; respecting the voluntarily expressed will of individuals as an intrinsic human right; openness to and acceptance of terminal sedation as a form of assisted dying; inclusion of assisted dying within the mandate and practice of palliative care.

The most prominent recommendation from declarations against euthanasia/assisted dying was for improvement in the provision of palliative care. This was followed by recommendations about access to and the administration of medications for adequate pain relief. They asserted that good palliative care and physical symptom control minimize the number of requests for hastened death and that governments should pay attention to lack of relevant health and social support, equality, and justice. Asserting that misconceptions about suffering at the end of life fuel the public demand for legalizing euthanasia, some declarations recommended public education about palliative care.

## Discussion

Our study has demonstrated that the practice of issuing declarations on euthanasia/assisted dying has emerged as a significant phenomenon within the field of end-of-life care. We have shown an increasing incidence of such declarations over time and their growing prominence as an advocacy tool. The declarations take specific (though varied) positions on the issue of legalization of euthanasia/assisted dying and aim to promote these to gain public support and/or favorable actions from governments. Despite their emerging significance, no commentary exists to our knowledge on such advocacy documents and their role in end-of-life debates and discourse. As the discussion on these issues spreads to more countries we are likely to see the appearance of further declarations of this type.

Our analysis shows a specific geographic range in the declarations identified. They all emanate from the United States, Canada, Western Europe, Australia, and New Zealand. These are countries where active measures have taken place to consider the value of legalizing euthanasia/assisted dying or where such legalization has already taken place. The absence of declarations from other parts of the word, including Asia and Africa is notable. Although discussions and studies exploring perceptions on the issue of euthanasia and assisted dying are emerging from these parts of the world (Saadery, 2014; Rao & Satyanarayana, 2016), relevant organizations from these countries have not yet prioritized declarations on the issue, or such organizations may not yet exist. It seems likely however that greater prominence will be given to debates about euthanasia/assisted dying in low- and middle-income countries and the appearance of such declarations from these settings is therefore to be expected.

The diversity of viewpoints on euthanasia/assisted dying is strikingly depicted in these declarations. Declarations for euthanasia/assisted dying range from those which endorse the decriminalization of assisted dying, to those which demand it as a fundamental human right. Declarations against range from those suggesting that assisted dying may not be the right solution to the problem of suffering, to others which strongly condemn initiatives to legalize euthanasia.

Although declarations for and against use some terminologies in common, the extent of their meaning and use differs significantly. Respecting the contents of a living will is a commonly recognized issue in end-of-life care. Yet although declarations favoring euthanasia extend the value of the living will to those expressing the wish to die, those against do not support its use to facilitate medical assistance to end life.

Although all declarations express their intention to promote dignified death, those for euthanasia consider respecting autonomous decisions of the individual on the timing, place, and manner of death as aspects of dignity. Declarations against euthanasia, however, present dignity as an equal and inviolable quality inherently possessed by human beings. They present the view that intentional killing of a human being, even at their voluntary request due to intractable suffering, undermines human dignity.

Despite their wide ranging characteristics and divided perspectives, euthanasia/assisted dying declarations share some of the wider principles of advocacy. They identify with disadvantaged populations, promote their cause, and invoke responses from positions of authority and professional groups, as well as from wider communities (Gray & Jackson, 2002; Price, 2003).

We acknowledge certain limitations to our study. Although the search for declarations was conducted in a systematic way, it is possible there may be other declarations we did not find, for example declarations could have used different terminology in their titles to our keywords or declarations may have been issued in other languages than English. Therefore, while capturing the landscape of declarations to a significant degree, there may be other declarations on euthanasia/assisted dying that are not covered in this study. We consider this a small possibility however. The findings of our study are also limited by the contents of these advocacy documents. We acknowledge that these may not necessarily represent the views of all individuals that make up these organizations, though they are the declared organizational position on the issue. It is also possible that there may be other organizations concerned about the legalization of euthanasia/assisted dying that have not considered it a high enough priority to issue a declaration.

Declarations relating to euthanasia and assisted dying represent the views and demands of diverse communities of interest concerned about suffering at the end of life, often with a determination to make their voices heard and to advocate for change. Our study has catalogued the emergence of this particular form of intervention as an advocacy tool in the wider debates about end-of-life issues. We have identified the various organizations involved, the positions represented and the recommendations made. In so doing, we have opened up a space for further analytic work and more comparative analysis of declarations across a range of end-of-life issues. Further exploration of these declarations in the light of their respective contexts will help understand their significance and impact.
